# Supplementary material for: A large-scale MIMO antenna system for 5G IoT applications
Source: PLoS One. 2024 Jan 22;19(1):e0292383. doi: 10.1371/journal.pone.0292383 (PMC10824423; doi:10.1371/journal.pone.0292383)
Supplement: Attachment — Submitted filename: Response to Reviewers.docx [file pone.0292383.s001.docx]

**Original Manuscript ID:** PONE-D-23-25130

**Original Article Title:** “A large-scale MIMO antenna system for 5G IoT applications”

**To:** Reviewer

**Re:** Response to reviewer

Dear Reviewer,

We appreciate you for your precious time in reviewing our paper and providing valuable comments. It was your valuable and insightful comments that led to possible improvements in the current version. The authors have carefully considered the comments and tried our best to address every one of them.

We are uploading our point-by-point response to the comments, an updated manuscript with red highlighting indicating changes, and a manuscript without track changes.

Best regards,

**Reviewer 1:** *The authors presented a closely spaced MIMO antenna and its mutual coupling reduction with the help of a half-wavelength microstrip line and a quarter-wavelength grounded stub. The manuscript is well-written and the data has been organized well. The presented results are quite interesting. However, the reviewer has some comments for the improvements.*

***Concern # 1:*** *What is the log tangent value of the antenna substrate? Did authors use the same substrate and thickness for the all MIMO designs?*

**Author response:** In this paper, the Taconic TLY-5 substrate is used for all designs. The loss tangent of this substrate is 0.0009.

**Author action:** The loss tangent is included in Paragraph 1, Section 3 of the revised manuscript.

***Concern # 2:*** *Comment on the measurement setup of the antenna.*

**Author response:** Agreed.

**Author action:** The measurement setup is added to Paragraph 1, Section 6 of the revised manuscript.

***Concern # 3:*** *Cite a proper reference for the equations used for the computation of the ECC/DG etc.*

**Author response:** Agreed.

**Author action:** The new references are added to the revised manuscript as ref [23].

***Concern # 4:*** *What is the unit of antenna profile in Table 1? Is it mm or the wavelength?*

**Author response:** The antenna’s profile in Table 1 is defined with respect to lambda.

**Author action:** The unit for the profile is updated in Table 1 of the revised manuscript.

***Concern # 5****:  The maximum isolation of the MIMO antenna is more than 25 dB as it is evident from the Figure 17. Why did the authors have stated maximum isolation as 20 dB in Table 1? Please explain.*

**Author response:** The authors would like to thank the Reviewer for pointing out our mistake. In fact, 20 dB is the isolation among all ports of the MIMO antenna at 4.8 GHz. The maximum isolation is 25 dB.

**Author action:** The maximum isolation is modified in Table 1 of the revised manuscript.

***Concern # 6:*** *Which shortwave was used for the simulation?*

**Author response:** The Ansys High-Frequency Simulation Structure (HFSS) is used in this paper.

**Author action:** The simulation tool is mentioned in Paragraph 4, Section 1 of the revised manuscript.

**Reviewer 2:** *The authors presented a low coupling MIMO antenna that can be easily extendable to a large-scale MIMO antenna system targeting 5G applications and IoT systems. The paper is well written, all the idea and concept is presented in professional way backed by strong mathematical explanation along with state-of-the-art.*

*There are few things that need to explain further:*

***Concern # 1:*** *Cite the reference from where the equations 1-3 are extracted.*

**Author response:** Agreed.

**Author action:** Ref [22] is added to the revised manuscript.

***Concern # 2:*** *Please comment on the bandwidth variation observed when the 2 x 2 MIMO array is extended to 2 X 3 MIMO array.*

**Author response:** Agreed.

**Author action:** Further discussion on the bandwidth variation is added to Paragraph 2, Section 5.2 of the revised manuscript.

***Concern # 3:*** *The decoupling is improved only at the desired bandwidth, what could be possible solution to improve the decoupling at the nearby frequency. Please comment.*

**Author response:** The authors would like to thank the Reviewer for the very constructive comment. In fact, achieving high isolation in a wide frequency range is a very challenging task. The authors are also working on that. The solution could be to produce additional resonances in the S_21_ profile.

***Concern # 4:*** *The authors should cite the proper reference from where the equation 4 and 5 are extracted.*

**Author response:** Agreed.

**Author action:** Ref [23] is added to the revised manuscript.

***Concern # 5****:  Since the work is focused on MIMO antenna, authors should add other MIMO performance parameters including Mean effective gain as explained in Highly selective multiple-notched UWB-MIMO antenna with low correlation using an innovative parasitic decoupling structure, 2023. Or the Channel capacity loss as explained in Self-decoupled tri band MIMO antenna operating over ISM, WLAN and C-band for 5G applications, 2023.*

**Author response:** Agreed.

**Author action:** The MEG and CCL are added to the revised manuscript as Fig. 21.

***Concern # 6:*** *Explain the reason of discrepancy among simulated and measured results of s-parameters. How it can be minimise?*

**Author response:** Agreed.

**Author action:** Further discussion is added to Paragraph 1, Section 6 of the revised manuscript.
